# Worked examples, cognitive aptitudes and the self‐explanation mechanism – A replication

**DOI:** 10.1111/bjep.70098

**Published:** 2026-05-22

**Authors:** Sarah Bichler, Matthias Stadler, Markus Bühner, Samuel Greiff, Frank Fischer

**Affiliations:** ^1^ Universität Passau Passau Germany; ^2^ Ludwig‐Maximilians‐Universität München München Germany; ^3^ Klinikum der Universität München Munich Germany; ^4^ School of Social Sciences & Technology and Centre for International Student Assessment (ZIB), Technical University Munich Munich Germany; ^5^ Present address: Ludwig‐Maximilians‐Universität München München Germany

**Keywords:** cognitive aptitudes, engagement activity, executive functions, problem‐solving, self‐explanation quality, worked examples

## Abstract

**Background:**

Self‐explaining is an engagement activity that supports learners' active use of instructional scaffolds such as worked examples. Self‐explanation quality is assumed to mediate the worked example effect on learning outcomes. However, prior investigations have relied on analytical approaches limited in scope and quality or have yielded inconclusive results

**Aims:**

We replicate a previous investigation that examines whether self‐explanation quality mediates the worked example effect in a worked examples – problem‐solving paradigm, while simultaneously considering key cognitive aptitudes (prior knowledge, working memory capacity (WMC), fluid intelligence, shifting ability) as moderating variables

**Method:**

We analysed self‐explanations from 115 university students solving six ill‐defined statistics problems with or without worked examples. Self‐explanation quality was defined as using specific case information from problem descriptions to justify statistical claims and coded by trained raters

**Results:**

Results showed a moderated mediation of the worked example effect via self‐explanation quality for prior knowledge and WMC, with stronger indirect effects at lower aptitude levels. The indirect effect was not moderated by fluid intelligence. No self‐explanation mediation emerged for shifting ability; shifting ability was confirmed as a moderator of the worked example effect, with stronger scaffold benefits at lower ability levels

**Conclusions:**

Whereas the original study found no evidence for self‐explanation quality as a mediator, the replication, drawing on stronger methodology, provides evidence for such mediation. However, this mechanism is not universal. Rather, worked examples exert their benefits either directly or via self‐explanation, contingent on learners' cognitive resources.

“To engineer situations as best we can based on what theoretical foundations tell us will promote good learning (…)” (Kolodner, 2004, p. 37) is a core goal in educational research and practice. Today, we can fall back on rich theoretical foundations and empirical evidence to do so. We know that learners benefit from scaffolding when learning something new and complex (Tabak & Kyza, 2018; Wood et al., 1976). For instance, worked examples – written step by step guides for how to solve a problem (e.g., Sweller & Cooper, 1985) – support learners from diverse populations learning a variety of tasks and skills across domains (Renkl, 2014; van Gog & Rummel, 2010, 2018). We also know that a learner's engagement with the to‐be‐learned material matters (Chi et al., 2018; Chi & Wylie, 2014). That is, a learner might not make active use of a provided worked example, lessening the impact this scaffold has on the learning outcome. Here, we can draw on a model for example‐based learning by Atkinson et al. (2000), who suggest that self‐explaining worked examples engages learners in the beneficial cognitive processes worked examples are designed to elicit.

Further, education researchers have identified many factors that influence learning outcomes, including cognitive aptitudes such as prior knowledge, general reasoning ability, and cognitive functions like working memory (e.g., Danili & Reid, 2004; Dochy et al., 1999; Primi et al., 2010; Tabak & Radinsky, 2014). More importantly, we know that these aptitudes interact with the instructional environments we design (Snow & Lohman, 1984; Sternberg et al., 1999). For instance, worked examples become less and less effective with a learner's increasing knowledge, ultimately being potentially harmful to learners who already gained domain expertise (Kalyuga, 2007; Kalyuga et al., 2003; Tetzlaff et al., 2025).

While self‐explaining is assumed to play a central role in securing the benefits of worked examples, this mediation assumption has rarely been rigorously tested. While prior research has extensively examined boundary conditions of the worked example effect, these have typically not been investigated in conjunction with learning process data. A recent study addressed this gap by applying a formal mediation framework while simultaneously accounting for cognitive aptitudes as moderators of the worked example effect (Bichler et al., 2022). However, this study did not find evidence for a mediating role of self‐explanation quality. To revisit this open question, the present study replicates this approach using an independent sample and an identical procedure. Specifically, we test whether self‐explanation quality mediates the effect of worked examples on learning gains in ill‐defined statistics problems, while considering key cognitive aptitudes as moderators of both direct and indirect effects.

## THEORETICAL AND EMPIRICAL FOUNDATIONS

### Scaffolding for learning: Worked examples

Scaffolds for learning are support tools provided to learners to help them perform aspects of tasks that are not yet within their reach without guidance (Pea, 2004; Tabak & Kyza, 2018; Wood et al., 1976). For instance, worked examples include a problem, solution steps, and the solution to the problem (e.g., Sweller & Cooper, 1985), a structure that “works out” demanding parts of a task for the learner to model after (van Gog & Rummel, 2018). With this design, worked examples focus learners on processing relations between problem states, operators, and structures, which learners often fail to do during unstructured problem‐solving (Sweller, 1988). During unguided problem‐solving, learners instead often fall back on weak strategies. These might help learners to eventually solve a problem, but it is unlikely that they support the abstraction of a cognitive schema for how to solve problems of the same or slightly different types. A worked example, in contrast, helps learners process relationships, domain rules or principles which supports the formation of schemas (Renkl, 2014; Sweller, 1988; Sweller et al., 2019). According to Cognitive Load Theory, a worked example does this by reducing element interactivity, as it “clearly indicates each problem state and the move required to reach the next state which is also depicted” (Sweller et al., 2024, p. 4). Hence, it reduces the intrinsic cognitive load solving the task imposes on the learner (Sweller, 2024). Because of this benefit, worked examples support expertise development, preparing learners for solving new problems in new contexts (Sweller, 1988; Sweller et al., 2019, 2024).

A large body of research attests to the worked example effect – studying examples has proven superior to problem‐solving for learners across age levels, disciplines, topics, and a range of outcomes (van Gog & Rummel, 2010, 2018). Further, worked examples are considered highly effective instructional support (Sweller et al., 2019) largely because researchers have ventured to explore and test the worked example effect's particularities.

### Engagement activity for learning: Self‐explaining

One of the key insights into the particularities of the worked example effect resulted from research that looked in detail at what learners actually do as they study examples. This body of work revealed that what distinguished effective and ineffective users of examples was that the former produced more, better‐structured, and more accurate explanations while studying the materials (Chi et al., 1989; Chi & VanLehn, 1991). We conceptualize self‐explaining, explanations directed at the learner themselves and generated while processing the learning materials (Roy & Chi, 2005), as an engagement activity for learning because it happens during the learning process and because it secures the learner's engagement with the provided scaffold. For example, self‐explaining a worked example secures active use of this scaffold (Renkl, 2014). Through this active use, learners are more likely to engage in the effective cognitive processes the worked example is designed to bring about: self‐explaining helps learners integrate new information into prior knowledge structures (Chi, 2000; Chi et al., 1994), detect gaps in their own understanding (VanLehn & Jones, 1993), and attend to structural features of examples (Rittle‐Johnson, 2006).

### The mediating effect of self‐explanation quality

Atkinson et al. (2000) describe that the positive effect of an example on the learning outcome is *transmitted* via self‐explanation quality. Studies reporting on this assumed mediation effect often compared how different self‐explanation prompts impact self‐explanation quality and in turn learning outcomes (Berthold et al., 2009; Berthold & Renkl, 2009; Hefter, 2021; Hefter et al., 2014, 2015) or how access vs. no access to instructional materials while self‐explaining examples impacts the quality of self‐explanations and in turn learning outcomes (Roelle & Renkl, 2020). These studies varied variables related to the engagement activity (self‐explanations), not the scaffold (worked examples).

Fewer studies investigated the mediation in the worked examples – problem‐solving paradigm, where the scaffold is varied (worked examples vs. problem‐solving) and the engagement activity is the same (all learners self‐explain). These studies found that learners produced higher‐quality self‐explanations in the worked example conditions than in conventional problem‐solving conditions (Schwonke et al., 2009; Stark et al., 2002) and reported higher learning outcomes in the worked example condition. However, they did not statistically test whether this worked example effect was mediated by self‐explanation quality.

### Cognitive aptitudes for learning

Beyond these insights into self‐explanation as an engagement activity potentially mediating the effect of worked examples on learning outcomes, research has also examined the conditions that determine for whom worked examples are most effective, shedding light on important boundary conditions of the effect. This line of research examined aptitude–treatment interactions (ATIs), which explore how the effectiveness of instructional scaffolds depends on learner characteristics (e.g., Snow & Lohman, 1984). According to aptitude theory, an instructional approach is effective only when it aligns with the learner's aptitudes (Cronbach & Snow, 1977; Snow, 1992; Sternberg et al., 1999). While most ATI studies have focused on cognitive aptitudes, the theory encompasses a broader range of learner factors, extending beyond cognitive dimensions (Shavelson et al., 2002; Snow, 1992).

#### Prior knowledge and general reasoning ability

In the context of worked examples, prior knowledge is arguably the most extensively studied and well‐supported cognitive aptitude influencing their effectiveness. The expertise reversal effect (Kalyuga, 2007) demonstrates that learners with lower domain knowledge benefit more from worked examples than from problem‐solving, whereas this pattern reverses for learners with higher prior domain knowledge (e.g., Bichler et al., 2020; Kalyuga, 2007; Tetzlaff et al., 2025).

Another cognitive aptitude that has received considerable attention in learning research is general reasoning ability, the capacity to reason and solve novel problems (Kriegbaum et al., 2018; Primi et al., 2010; Roth et al., 2015; Yuan et al., 2006). In fact, it was the most frequently investigated cognitive aptitude in early ATI research (e.g., Snow & Lohman, 1984), with evidence suggesting that learners with higher reasoning abilities tend to perform better in less structured environments, whereas learners with lower reasoning abilities tend to benefit more from highly structured instruction (e.g., Snow, 1992). This pattern has led researchers to investigate whether general reasoning ability (fluid intelligence, Gf) also moderates the worked example effect. However, the available evidence is mixed. One study found that the benefit of worked examples was strongest for learners with lower reasoning ability (Schwaighofer et al., 2016), whereas another failed to replicate this moderation effect (Bichler et al., 2020).

#### Working memory capacity

Yet another aptitude relevant for learning is working memory (WM) (Yeniad et al., 2013). WM is the mental workspace in which information can be stored, maintained, and manipulated (Miyake et al., 2000). Working memory is defined by limited storage capacity and consequently, it is often assessed in terms of this capacity (Draheim et al., 2016). Working memory occupies a prominent position in instructional theory (Sweller, 2004, 2010; Van Merrienboer & Sweller, 2005). Its limited storage capacity is often highlighted as the reason why instructional designs are (in)effective (e.g., Sweller et al., 2019). As worked examples are designed to reduce the cognitive demand on learners, it was hypothesized that “worked examples (…) may be even more effective for learners who have lower working memory capacity” (van Gog & Rummel, 2010, p. 160). However, empirical evidence does not support this hypothesis (Bichler et al., 2020; Schwaighofer et al., 2016). In real‐world learning situations, learners may naturally find ways to offload task demands (de Jong, 2010; Schwaighofer et al., 2016), worked examples may not compensate for lower WM capacity (Bichler et al., 2020), or individual differences in WM capacity become negligible during complex learning when cognitive demand is high, even for those who have more WM capacity (Paas & Sweller, 2014).

#### Shifting ability

In contrast to working memory capacity (WMC), shifting ability, another core executive function relevant to learning (Yeniad et al., 2013), has received little attention in instructional theory and research on instructional effectiveness. Shifting is the ability to quickly and flexibly switch between executing different operations or tasks (Diamond, 2013; Miyake et al., 2000): for example, holding a phone conversation while attending to something cooking on the stove, handing your little child a toy they are asking for, and returning to the discussion with the person on the other end of the line.

As conventional problem‐solving requires learners to frequently shift between problem states, different operators, and the goal state (Sweller et al., 2024), it was hypothesized that worked examples lower the shifting demand and are thus particularly effective for learners with lower shifting ability (Schwaighofer et al., 2016). Indeed, evidence showed that learners with lower shifting ability benefited more from worked examples than learners with higher shifting ability (Bichler et al., 2020; Schwaighofer et al., 2016).

### The three components of the instructionally supported learning framework

Motivated by the goal of examining rarely studied moderators such as fluid intelligence and executive functions, Schwaighofer et al. (2016) had students study statistics using ill‐defined problems in a worked example or a conventional problem‐solving condition. In both groups, students wrote down their solutions and justifications, thereby generating self‐explanations. The analysis focused on aptitude‐based boundary conditions, as developing a theoretical argument for understudied aptitudes warranted a dedicated investigation, leaving the question of self‐explanation mediation unaddressed. Bichler et al. (2022) later addressed this open question. Importantly, they did not only analyze the self‐explanation data but adopted an integrative analytical framework that simultaneously considered self‐explanation quality as a potential mediator and cognitive aptitudes as moderators of both direct and indirect effects.

Self‐explanation quality was operationalized as the extent to which learners' written solutions contained justified claims, that is, explicit connections between conceptual knowledge and case‐specific information. Applying a conditional process model that incorporates the scaffold, the engagement activity, and cognitive aptitudes simultaneously (Hayes, 2022), Bichler et al. (2022) found that although the worked example group outperformed the problem‐solving group on posttest performance, this advantage was not mediated by self‐explanation quality. Furthermore, the indirect effect did not vary as a function of prior knowledge, fluid intelligence, WMC, or shifting ability.

These findings suggest that the advantage of worked examples in this context cannot be attributed to higher‐quality self‐explanations but may instead arise from other mechanisms inherent to worked examples themselves. Bichler et al. (2022) proposed that for ill‐defined problems, the primary learning benefit lies in deriving a solution strategy that helps to systematically break down the task into manageable steps. This process appears more crucial for ill‐defined problems, which lack a clear solution procedure, than the establishment of connections between conceptual knowledge and case information (self‐explanation quality was defined as the learner's ability to map theoretical concepts onto specific case information and provide justifications for statistical choices).

An alternative, yet related, explanation for why both groups produced similar quality self‐explanations despite the worked example group outperforming the problem‐solving group is that learners in the worked example condition engaged in analogical reasoning. By mapping relevant elements from the worked examples onto the novel problems in the posttest, these learners likely compared relational structures between the practice tasks and the new, slightly different problems. This process of analogical transfer, which learners in the problem‐solving condition may not have been adequately prepared for, could account for the superior performance observed (Reimann & Schult, 1996).

## THE PRESENT STUDY

In this study, we address the open question of whether self‐explanation quality mediates the effect of worked examples on learning gains in the worked examples–problem‐solving paradigm. This question is central to theories of example‐based learning, which posit self‐explanation as a key mechanism underlying the effectiveness of worked examples (Atkinson et al., 2000), yet empirical evidence remains inconclusive (Bichler et al., 2022). To investigate this mechanism, we apply the analytic approach proposed by Bichler et al. (2022) to data collected in Bichler et al. (2020), which itself constitutes a replication of Schwaighofer et al. (2016) (see Figure 1 for a visualization of the study lineage). Although self‐explanation data were collected in the 2020 study, they were not analyzed at the time, as the investigation, like the original study, focused exclusively on aptitude‐based boundary conditions of the worked example effect.

**FIGURE 1:**
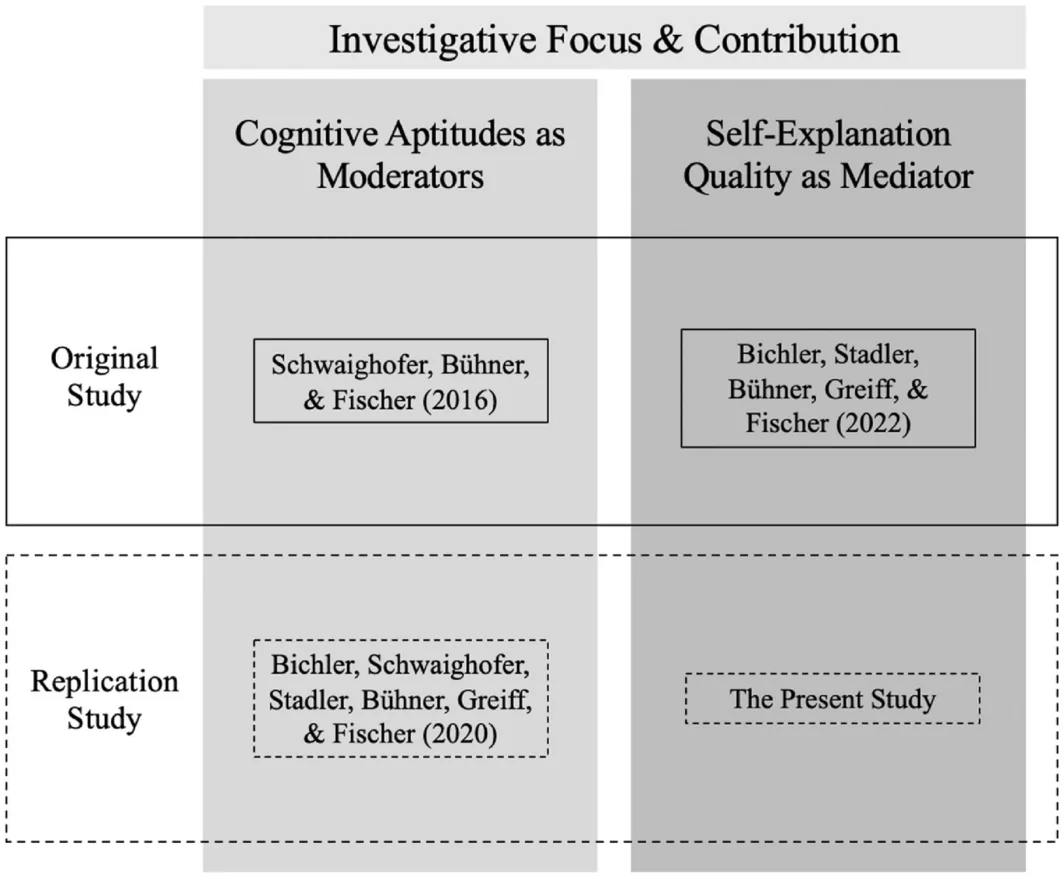
Overview of study lineage and replication structure. The figure illustrates the relationship between the original study (Bichler et al., 2022; Schwaighofer et al., 2016) and the replication study (Bichler et al., 2020 and the present study). Schwaighofer et al. (2016) and Bichler et al. (2020) focused on aptitude‐based boundary conditions of the worked example effect. Self‐explanation data collected in that study were not analysed at the time. Bichler et al. (2022) addressed the mediation question using a moderated mediation framework while accounting for cognitive aptitudes. The present study applies the same analytical approach as Bichler et al. (2022) to the independent replication dataset, extending the investigation of the self‐explanation quality mediation assumption.

The present study addresses the self‐explanation mediation question by analysing previously unexamined self‐explanation data from the replication study (Bichler et al., 2020). Following the holistic approach proposed by Bichler et al. (2022), our study involves partial re‐analyses of the moderation‐focused replication to obtain robust estimates of the mediation effect, as ignoring established moderators may bias mediation estimates when indirect effects depend on learner characteristics. Thus, the present study extends Bichler et al. (2020) by incorporating newly coded process data (learners' self‐explanations) to rigorously test the mediation hypothesis in the worked examples–problem‐solving paradigm.

We test four conditional process models (Figure 2) to address the following research questions: Is the effect of worked examples on learning gains mediated by self‐explanation quality, and is the indirect or any other effect in the model dependent on prior knowledge, shifting ability, WMC, and fluid intelligence?

**FIGURE 2:**
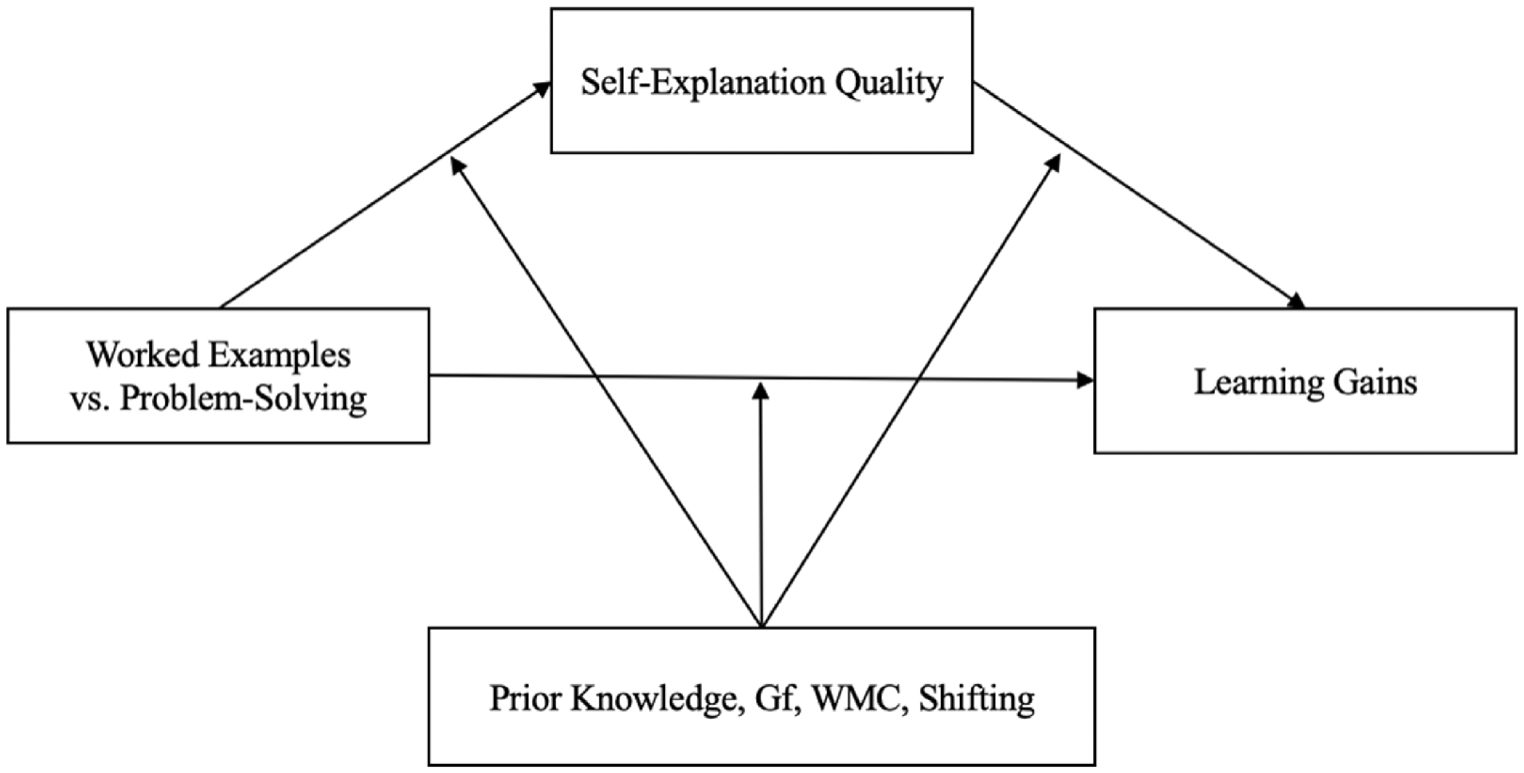
Three components of instructionally supported learning model. Conditional process model assuming the worked example effect on learning gains is transmitted by the quality of self‐explanations and testing whether any effect in the model including the indirect effect is contingent on levels of prior knowledge, Gf = fluid intelligence, WMC = working memory capacity, or shifting ability. Model 59 in PROCESS (Hayes, 2018).

By addressing the same research questions, using the same analytic approach, and testing the same moderated mediation models, we consider the present study a replication of Bichler et al. (2022) that helps to discern genuine instructional effects from study‐specific findings and to strengthen the theoretical and empirical foundations (Bauernfeind, 1968; Perry et al., 2022; Plucker & Makel, 2021) on which learning environments are built. We specifically address the discrepancy between theory, which posits self‐explanation as a core mechanism of example‐based learning (Atkinson et al., 2000), and prior empirical results, which did not support mediation in the worked example – problem‐solving paradigm (Bichler et al., 2022). This allows us to evaluate whether the absence of the mediation in the original study reflects a false negative, methodological constraints, or whether the theoretical assumption about self‐explanation as a universal mediator of worked example effects requires adjustment.

## METHOD

### Data source

The present study draws on data from Bichler et al. (2020). We therefore describe the design and procedures of that study, supplemented with details not previously reported regarding the self‐explanation data. The analytic approach we applied follows Bichler et al. (2022); any deviations from the original investigation are explicitly noted. Ethics approval was obtained for the (re‐)use of the Bichler et al. (2020) dataset.

### Participants and design

We used data from *N* = 115 first to twelfth semester university students who participated in the Bichler et al. (2020) study. Most participants identified as women (86%) with a mean age of 23.34 years (SD = 5.03) and participation was compensated with 55€ or a participation certificate relevant for obtaining a degree in Psychology. Participants were randomly assigned to a worked examples (*n*_1_ = 57) and a problem‐solving condition (*n*_2_ = 58).

### Materials

Participants studied materials on the General Linear Model and solved six ill‐defined statistical problems in 45 min. Participants scrolled through the materials on a laptop computer, and a pencil and paper were available to write self‐explanations for the six problems. Each statistical problem describes the research interest of a fictitious researcher, including relevant and irrelevant information and ending with the question of how the described research problem could be addressed statistically.

### Experimental manipulation

For each of the six ill‐defined problems, the worked‐out solution was presented across three pages, with each page modelling one of the three solution steps: (1) identify variables and design, (2) identify an appropriate statistical analysis, and (3) describe the applicable statistical assumptions. The targeted solution step appeared at the top of the page, while the remainder of the page displayed the problem description. Relevant case information was highlighted using colour‐coding and visually linked to text boxes that illustrated how theoretical concepts were applied to the case information (Figure 3). Participants in the problem‐solving condition used the same learning materials but had access only to the problem descriptions (six pages with one problem description on each).

**FIGURE 3:**
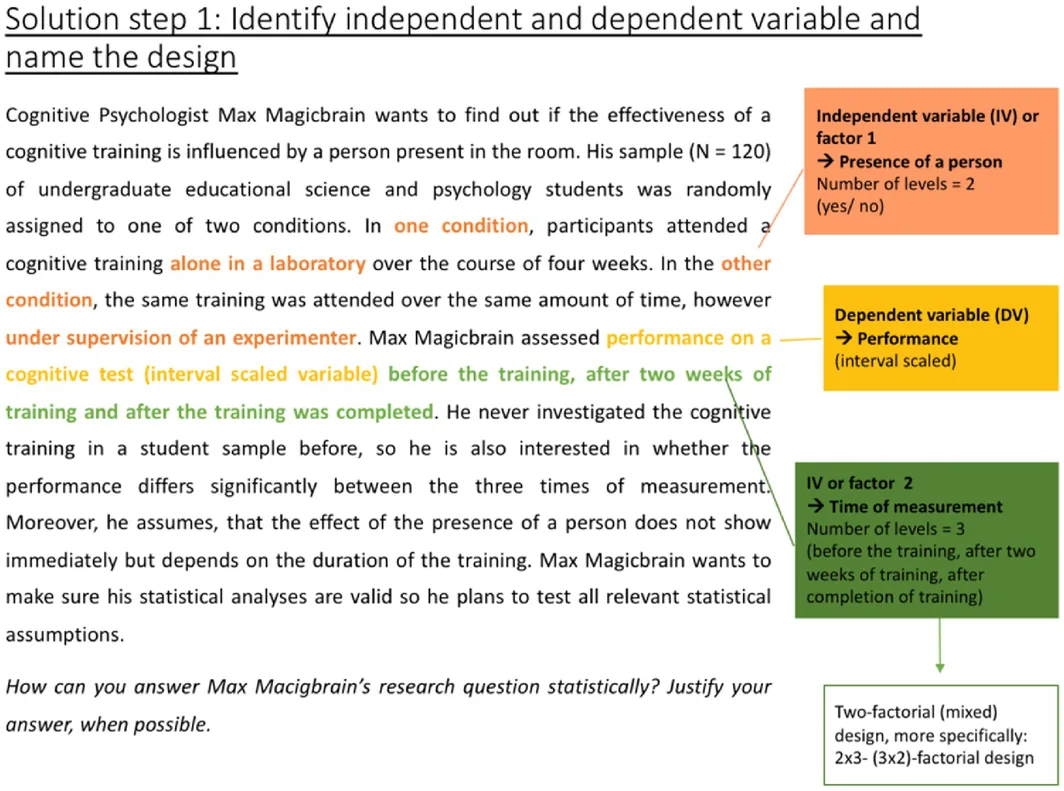
Worked example. Worked example: Modelling solution step 1 “Identify variables and design” for one of the six problems. Relevant information is bolded and colour‐coded. Arrows and text boxes with the same colour‐coding show how specific problem information relates to relevant statistical concepts.

### Self‐explanation quality

Participants generated self‐explanations for each problem while solving it, in response to a prompt that asked them to justify their solutions. Self‐explanation quality was assessed using the coding scheme developed by Bichler et al. (2022). The authors used three statistical problems. The replication study used six problems to better approximate an authentic learning session and to strengthen the self‐explanation quality measure. For the three additional problems, we developed new coding schemes, equivalent to the existing ones.

In line with the prompt given to participants, self‐explanation quality was operationalized as the extent to which learners produced justified claims. Claims referred to identifying relevant conceptual information for a problem (e.g., one predictor, one control, one outcome variable). Justifications referred to applying these concepts to specific case information in the problem (e.g., “The predictor is prior math knowledge [low vs. high], and the control variable is intelligence”).

For each problem, learners could make up to three claims, corresponding to the three solution steps modelled in the worked examples (identifying variables and design, selecting a statistical analysis, stating applicable statistical assumptions). Each claim was first coded as complete, partial, or absent/inaccurate. Next, its justification was coded as fully, partially, or not applied to the case information. Because participants were explicitly prompted to justify their answers, greater value was placed on complete justifications than on complete claims without justification. The scoring scale reflects this emphasis, ranging from 3 points (complete claim, fully justified) to 2.5 points (incomplete claim, fully justified), down to 0 points (“no claim, not justified”) (Table 1). A maximum of 3 points per claim was possible, yielding up to 9 points per problem. We summed scores across all 18 claims from the six problems to create each participant's self‐explanation quality score. The internal consistency of this scale was good (Cronbach's *α* = .78).

**TABLE 1:** Self‐explanation quality indicator.

Claim (concepts)		Justification (application to case information)	Score
Complete	+	Fully applied	3
Partial	+	Fully applied	2.5
Complete	+	Partially applied	2
Partial	+	Partially applied	1.5
Complete	+	Not applied	1
Partial	+	Not applied	.5
Absent/inaccurate	+	Not applied	0

*Note*: Three claims per problem (0–9 points) aggregated across 6 problems resulting in a self‐explanation quality indicator ranging from 0 to 54.

Two researchers trained coding on a few examples and then independently coded 10% of the material. Cohen's *κ* was calculated as a measure of interrater reliability, disagreements were discussed, the coding rules refined, and the procedure repeated until we achieved at least *κ* > .6 for each code. Across all six problems, two to five rounds of independent coding were needed to achieve a minimum Cohen's *κ* = .6 on all codes. After reaching interrater reliability, the first author coded all self‐explanations.

### Application‐oriented knowledge

Application‐oriented knowledge in statistics was assessed with six open response questions before and after the learning phase. Each item described a statistical problem similar to but shorter than the practice problems. Participants were asked to answer three sub‐questions, of which each one pertained to one of the three solution steps modelled in the worked example. Responses for pre‐ and posttest were coded with a rubric and a maximum score of 26 points. We used the pretest sum score as a prior knowledge indicator (moderating variable). We used the difference between post‐test and pretest scores (gains) as a dependent variable in all analyses unless indicated otherwise. Bichler et al. (2020) reported good reliability for the knowledge measure.

### Cognitive aptitudes (moderating variables)

We briefly summarize the assessment of cognitive aptitudes which is reported in detail in Bichler et al. (2020). Working memory capacity (WMC) was measured with the shortened version of the automated operation span task, symmetry span task, and reading span task (Oswald et al., 2015). Shifting ability was measured with the colour‐shape task, number‐letter task, and category‐switch task (Friedman et al., 2016), and fluid intelligence with the adaptive version of the Intelligence structure Battery (INSBAT, Arendasy et al., 2012). All tasks were administered on a laptop. While the fluid intelligence measure and shifting ability assessment showed good reliabilities, the working memory capacity measure yielded mixed reliability results for the three complex span tasks.

### Procedure

Participants completed demographics, the pretest, and the cognitive aptitudes test battery in a first session. In a second session, they worked on the practice problems and completed the posttest. Both sessions took place in a typical seminar room of the university with a researcher present.

### Statistical analysis

Conditional process analyses were run with the SPSS macro PROCESS (IBM SPSS Statistics, Version 24) written by Hayes (2018). We estimated the effects of the independent, mediating, and moderating variables as well as their interactions (Figure 1; Model 59). We used a 5% alpha level, reported unstandardized effect sizes and 95% bootstrapped confidence intervals (5000 samples) (CI_boot_). We reported one‐tailed *p‐*values and estimated the 90% two‐tailed CI_boot_ as an approximation for the 95% one‐tailed CI_boot_ for effects for which prior evidence or theory indicates directional hypotheses. This is clearly marked in the results section. We used age and semester as covariates to maintain consistency with the original study (Bichler et al., 2022). A histogram of self‐explanation quality shows that the mediator variable is approximately normally distributed. Boxplots showed no outliers for self‐explanation quality.

## RESULTS

### Descriptive statistics and correlations

Participants in the worked example condition (*n*_1_ = 57) had descriptively higher self‐explanation quality (*M* = 15.89, SD = 10.06) than participants in the problem‐solving condition (*n*_2_ = 58, *M* = 13.59, SD = 6.74). Correlations of the mediator and other study variables are presented in Table 2.

**TABLE 2:** Correlations for self‐explanation quality with control and moderating variables.

		Control variables		Moderating variables			
		Age	Semester	Prior knowledge	WMC	Shifting	Gf
Self‐explanation quality	*r* *p*	−.19 .038	.23 .016	.29 .002	−.01 .897	−.14 .150	.17 .062

Abbreviations: WMC, working memory capacity; Gf, fluid intelligence; note that higher scores for shifting indicate lower ability and lower scores indicate higher ability.

### Self‐explanation mediation with prior knowledge as moderator

In the first model, we tested the self‐explanation quality mediation and its contingency on prior knowledge (Figure 4). Results show an indirect effect of worked examples through self‐explanation quality on posttest knowledge. The indirect effect is contingent on levels of prior knowledge (a moderated mediation). More specifically, the indirect effect becomes weaker with increasing levels of prior knowledge. Note that the indirect effect is not statistically significant at higher prior knowledge levels (Table 3). This means that a learner with average prior knowledge in the worked example group is estimated to reach .43 more points (score about 2% higher) in the posttest compared to a learner with average prior knowledge in the problem‐solving group and that this difference is due to worked examples supporting learners to generate self‐explanations of higher quality, which in turn affects their learning gain (Hayes, 2018, p. 84).

**FIGURE 4:**
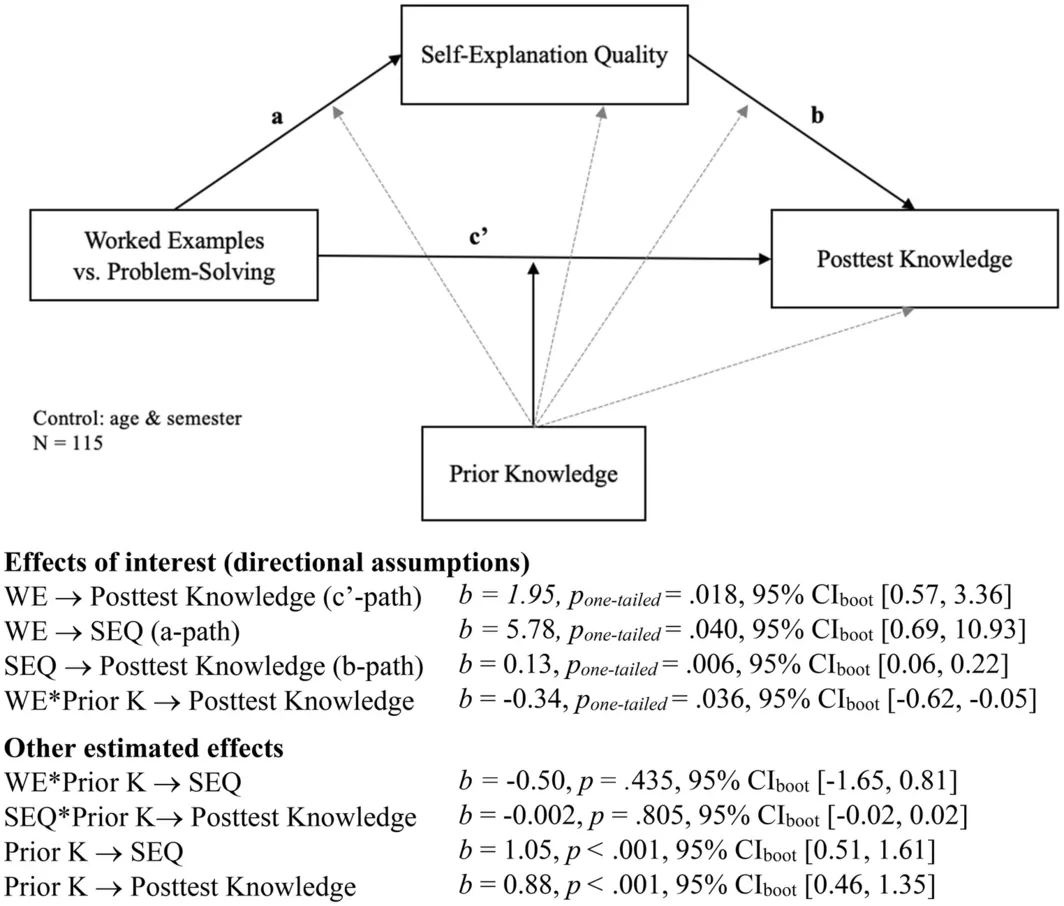
Conditional process model with prior knowledge as moderator. WE = worked examples vs. problem‐solving; SEQ = self‐explanation quality; Prior K = prior knowledge; * = interaction effect; c'‐path = direct effect (not total effect); indirect effect on different levels of prior knowledge is reported in a separate table. PROCESS model 59: Conditional process model including one mediator (SEQ) and one moderator (prior knowledge) (Hayes, 2018); for directional tests, we report the 90% two‐tailed CI_boot_ as an approximation for the 95% one‐tailed CI_boot_ (bootstrapped confidence interval).

**TABLE 3:** Indirect effect of worked examples vs problem‐solving on posttest knowledge through self‐explanation quality for different levels of the moderator prior knowledge.

Prior knowledge	*b*	SE_boot_	95% CI_boot_
Mean − 1 SD = 1.58	.63	.318	.04, 1.31
Mean = 4.29	.43	.210	.09, .91
Mean + 1 SD = 7.01	.25	.302	−.22, .99

*Note*: *N* = 115. Prior knowledge is measured as points in test.

Abbreviation: CI_boot_, bootstrapped confidence interval.

The conditional process analysis reproduces the interaction effect of worked examples and prior knowledge reported by Bichler et al. (2020), who did not model the self‐explanation quality mediation (Table 4).

**TABLE 4:** Conditional effect of worked examples vs problem‐solving on posttest knowledge for different levels of the moderator prior knowledge.

Prior knowledge	*b*	*p*_one‐tailed_	LLCI	ULCI
Mean − 1 SD = 1.58	1.42	.021	.27	2.57
Mean = 4.29	.50	.155	−.32	1.32
Mean + 1 SD = 7.01	−.41	.284	−1.60	.78

*Note*: The 90% two‐tailed CI is estimated as an approximation of the 95% one‐tailed CI. Prior knowledge is measured as points in test.

Abbreviations: LLCI, lower level confidence interval; ULCI, upper level confidence interval.

### Self‐explanation mediation with shifting ability as moderator

The second moderated mediation model includes shifting ability as a moderating variable (Figure 5). In this model, the indirect effect of worked examples through self‐explanation quality is not statistically significant on any level of the moderator (Table 5). Reproducing the effect reported by Bichler et al. (2020), worked examples are more effective than problem‐solving for learners with lower shifting ability, and their effectiveness decreases with increasing shifting ability (note that higher values for shifting indicate *lower* ability) (Table 6).

**FIGURE 5:**
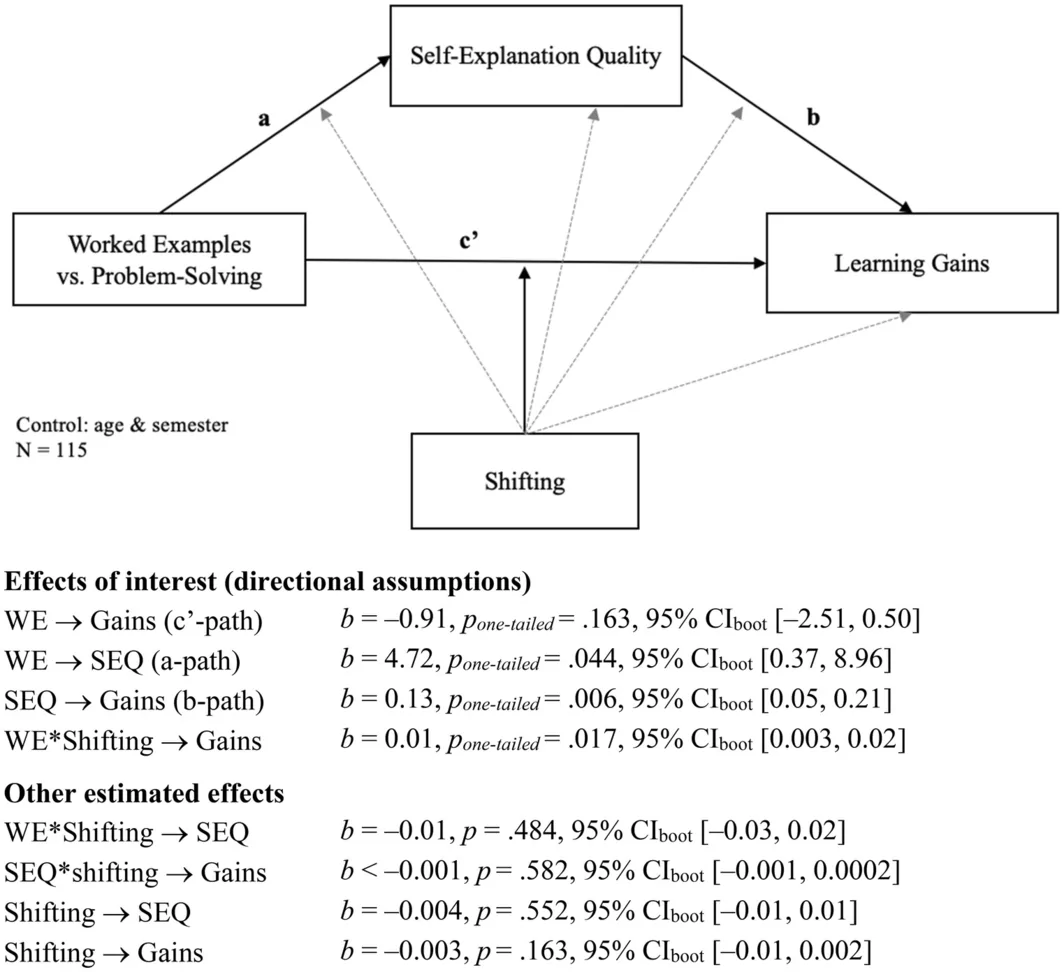
Conditional process model with shifting ability as moderator. WE, worked examples vs. problem‐solving; SEQ, self‐explanation quality; *, interaction effect; c‐path, direct effect (not total effect); indirect effect on different levels of shifting is reported in a separate table. PROCESS model 59: Conditional process model including one mediator (SEQ) and one moderator (shifting) (Hayes, 2018); for directional tests, we report the 90% two‐tailed CI_boot_ as an approximation for the 95% one‐tailed CI_boot_ (bootstrapped confidence interval).

**TABLE 5:** Indirect effect of worked examples vs problem‐solving on gains through self‐explanation quality for different levels of the moderators.

Moderator	Levels of moderator	*b*	SE_boot_	95% CI_boot_
Shifting ability	Mean − 1 SD = 37.88	.53	.387	−.04, 1.47
	Mean = 183.77	.33	.204	−.01, .80
	Mean + 1 SD = 329.66	.16	.222	−.25, .66
Working memory capacity	Mean − 1 SD = .55	.56	.337	.04, 1.35
	Mean = .69	.25	.155	.01, .60
	Mean + 1 SD = .83	.05	.166	−.21, .48
Fluid Intelligence	Mean − 1 SD = −.56	.15	.196	−.18, .60
	Mean = .26	.26	.170	.01, .66
	Mean + 1 SD = 1.09	.34	.310	−.16, 1.07

*Note*: Shifting measure = switch costs, mean reaction times in milliseconds; working memory capacity measured as mean of proportion of correctly recalled items; raw scores for fluid intelligence.

Abbreviation: CI_boot_, bootstrapped confidence interval.

**TABLE 6:** Conditional effect of worked examples vs problem‐solving on learning gains for different levels of the moderators.

Moderator	Levels of moderator	*b*	*p*_one‐tailed_	LLCI	ULCI
Shifting ability	Mean − 1 SD = 37.88	−.57	.237	−1.90	.75
	Mean = 183.77	.71	.090	−.16	1.59
	Mean + 1 SD = 329.66	2.00	.007	.68	3.32
Working memory capacity	Mean − 1 SD = .55	.25	.340	−.75	1.25
	Mean = .69	.78	.054	−.02	1.59
	Mean + 1 SD = .83	1.32	.045	−.04	2.60
Fluid Intelligence	Mean − 1 SD = −.56	.90	.070	−.10	1.90
	Mean = .26	.91	.042	.05	1.77
	Mean + 1 SD = 1.08	.92	.107	−.30	2.13

*Note*: The 90% two‐tailed CI is estimated as an approximation of the 95% one‐tailed CI. Shifting measure = switch costs, mean reaction times in milliseconds; working memory capacity measured as mean of proportion of correctly recalled items; raw scores for fluid intelligence.

Abbreviations: LLCI, lower level confidence interval; ULCI, upper level confidence interval.

### Self‐explanation mediation with WMC as moderator

The third conditional process analysis includes working memory capacity (WMC) as moderator (*N* = 114 due to one missing value for WMC) (Figure 6). The indirect effect of worked examples through self‐explanation quality is dependent on WMC. Worked examples support higher quality self‐explanations, which in turn are associated with higher learning gains, an effect that is stronger for learners with lower WMC as its strength decreases with increasing WMC. However, the indirect effect was not statistically significant for learners with high WMC (Table 5). There was no statistically significant interaction of worked examples and WMC (Table 6), this reproduces the results reported by Bichler et al. (2020).

**FIGURE 6:**
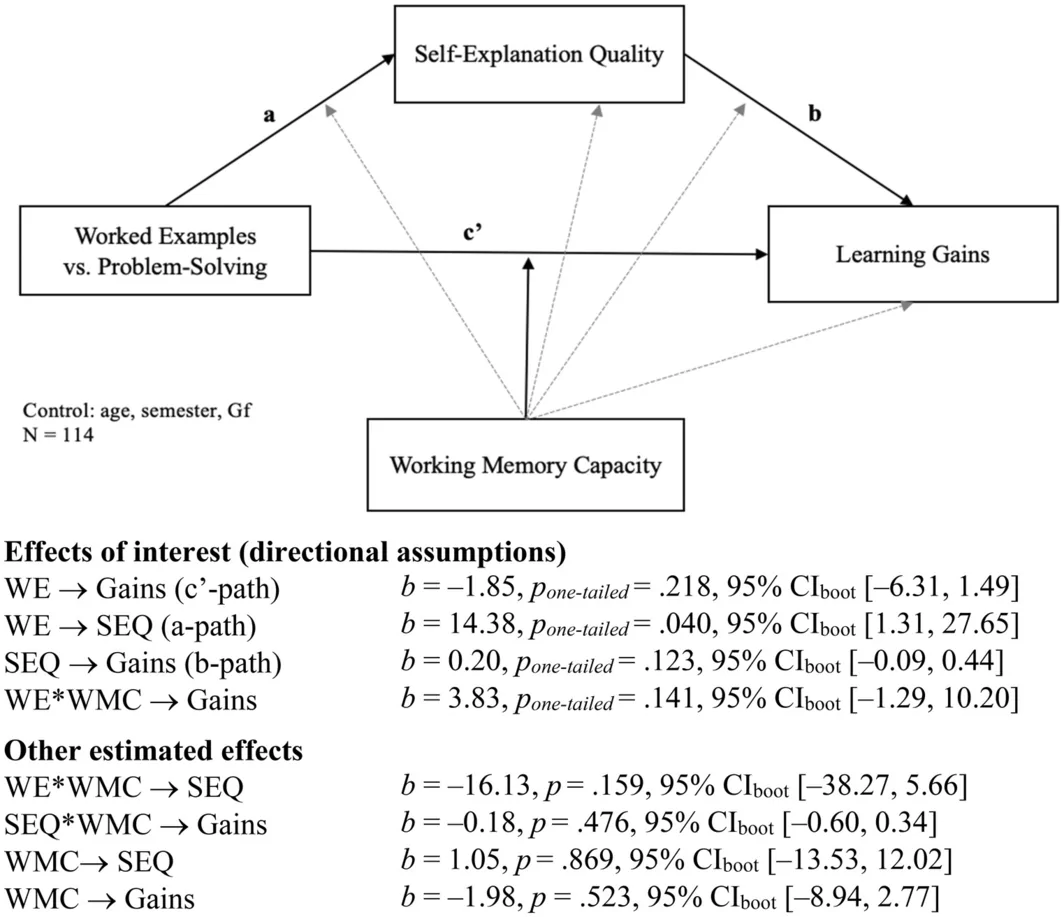
Conditional process model with working memory capacity as moderator. WE, worked examples vs. problem‐solving; SEQ, self‐explanation quality; WMC, working memory capacity; Gf, fluid intelligence; *, interaction effect; c‐path, direct effect (not total effect); indirect effect on different levels of shifting is reported in a separate table. PROCESS model 59: Conditional process model including one mediator (SEQ) and one moderator (WMC) (Hayes, 2018); for directional tests, we report the 90% two‐tailed CI_boot_ as an approximation for the 95% one‐tailed CI_boot_ (bootstrapped confidence interval).

### Self‐explanation mediation with fluid intelligence as moderator

The fourth moderated mediation model includes fluid intelligence as a moderating variable (*N* = 114 because WMC is included as a control variable) (Figure 7). The indirect effect of worked examples through self‐explanation quality is only statistically significant for the mean level of fluid intelligence. Thus, there is an indirect effect, and we did not obtain evidence that this effect is contingent on levels of fluid intelligence (Table 5). Fluid intelligence does not moderate the direct effect of worked examples on learning gains (Table 6), this reproduces the results reported by Bichler et al. (2020).

**FIGURE 7:**
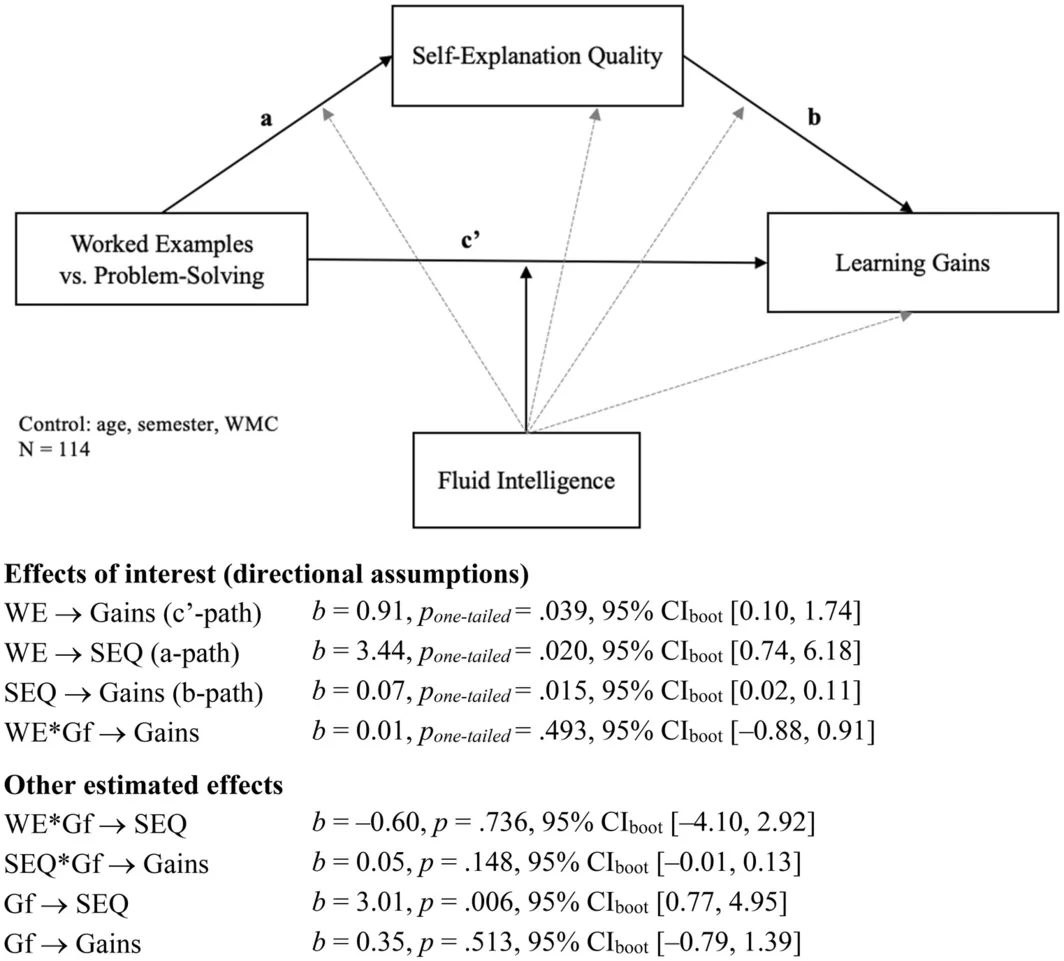
Conditional process model with fluid intelligence as moderator. WE, worked examples vs. problem‐solving; SEQ, self‐explanation quality; Gf, fluid intelligence; WMC, working memory capacity; *, interaction effect; c‐path, direct effect (not total effect); indirect effect on different levels of shifting is reported in a separate table. PROCESS model 59: Conditional process model including one mediator (SEQ) and one moderator (Gf) (Hayes, 2018); for directional tests, we report the 90% two‐tailed CI_boot_ as an approximation for the 95% one‐tailed CI_boot_.

## DISCUSSION

We argue that learning outcomes result from a complex interplay between instructional scaffolds, engagement activities, and cognitive aptitudes. While these three components have been studied, the mediating role of self‐explanation quality in the worked example–problem‐solving paradigm remains underexamined, and joint analyses of mediators and moderators are rare. To address this, we replicated Bichler et al.'s (2022) investigation of self‐explanation quality as a mediator of the worked example effect and its dependence on cognitive aptitudes as moderators of the assumed indirect effect.

Building on Atkinson et al.'s (2000) framework, we hypothesized that learners in the worked example condition would acquire more knowledge than those in the problem‐solving condition due to their tendency to produce higher‐quality self‐explanations. Acknowledging that the worked example effect is influenced by learners' cognitive aptitudes, we incorporated prior knowledge, shifting ability, working memory capacity (WMC), and fluid intelligence as moderators to examine how these factors interact with the mediation effect.

We tested four conditional process models, one for each moderating variable. We found evidence for an indirect effect of worked examples through self‐explanation quality on application‐oriented knowledge (gains) in three of those models: prior knowledge, WMC, and fluid intelligence. We found a dependency of this indirect effect on levels of prior knowledge and WMC. This contrasts with Bichler et al. (2022), who did not find evidence for the mediation or its contingencies. Our results align with the original study with respect to the model including shifting ability, where both studies detect no evidence for the mediation. These result patterns highlight three important aspects to discuss: First, how do we interpret the mixed findings of the original and replication studies? Second, how do we interpret the fact that there is evidence of an indirect effect in the presence of some but not all moderating variables? And third, how do we interpret the contingencies that we found?

### Mixed findings across original and replication studies

Considering that there is not yet a systematic investigation of self‐explanation quality as mediator in the worked examples – problem‐solving paradigm, the original and our replication study seem to offer competing explanations. We focus on two key differences between the original study (Bichler et al., 2022) and our replication, the sample size and the self‐explanation quality measure, to make sense of the contrasting findings. While Bichler et al. (2022) used data from *N* = 74 participants, we report our results on a basis of *N* = 115 participants. While our sample is still not big enough to reach desired statistical power (Aguinis et al., 2005; Schoemann et al., 2017; Thoemmes et al., 2010), our study has higher statistical power to detect the indirect effect. Further, participants in our study solved 6 in comparison to 3 practice problems in the original study (Bichler et al., 2022). Thus, our study employed a more robust measure of the mediator. Due to these improvements and the strong theoretical considerations (Atkinson et al., 2000), we place greater confidence in the results of the replication study, supporting the hypothesized indirect effect. However, we also recognize the need for further replications to strengthen the credibility of this evidence, including the possibility that future replications may not support the self‐explanation mediation effect. Such outcomes could lend credence to alternative explanations, such as a direct effect of worked examples (Bichler et al., 2022; Rittle‐Johnson, 2006; Sweller, 1988) or the role of analogical reasoning (Reimann & Schult, 1996).

### No self‐explanation quality mediation with shifting as moderator

Our study provides evidence of an indirect effect of worked examples through self‐explanation quality in all models except when shifting ability was the moderator. This raises the question of whether there is a specific match between worked examples and shifting ability. Notably, the self‐explanation mechanism was strongest in learners with lower levels of other cognitive aptitudes, and worked examples have been shown to be most effective for learners with lower shifting ability (Bichler et al., 2020; Schwaighofer et al., 2016). This suggests that the worked example itself may reduce the shifting demands of the task, rather than the combination of worked example and self‐explanation. Learners with lower shifting ability might not need an engagement activity like self‐explaining to benefit from a support tool if that tool is designed to directly match their cognitive needs (Snow, 1992). By demonstrating how relevant concepts apply to a given problem, how these concepts interrelate, and what steps are needed to solve an ill‐defined problem (Bichler et al., 2022), a worked example may be uniquely positioned to match the cognitive needs of learners who struggle to switch between conceptual and problem information, which alone helps learners benefit from the support provided.

### Dependencies of the indirect effect on cognitive aptitudes

The indirect effect of worked examples through self‐explanation quality was strongest for learners with less prior knowledge and became weaker with increasing prior knowledge. Notably, the indirect effect did not exist for high levels of prior knowledge. As learners with less prior knowledge lack understanding of domain concepts, self‐explaining might have supported them to use the worked examples to identify their knowledge gaps (Roy & Chi, 2005; VanLehn & Jones, 1993) and to integrate their rather fragmented understanding (Chi, 2000). While our results indicate that the advantage of worked examples over problem‐solving diminishes with increasing prior knowledge, a pattern consistent with the expertise‐reversal effect (Kalyuga, 2007), they do not clarify whether worked examples and problem‐solving are equally effective for more knowledgeable learners, or if problem‐solving may be more advantageous. Similarly, as the indirect effect weakens with higher prior knowledge, it remains uncertain whether adding engagement activities is more or less beneficial than using worked examples or problem‐solving alone for these learners.

Similarly, the indirect effect was strongest for lower WMC levels, became weaker with increasing WMC levels, and was not statistically significant for high WMC levels. Self‐explaining the example particularly helped those learners who cannot easily maintain and manipulate multiple elements in working memory to focus on each statistical concept and its corresponding case information step‐by‐step. By externalizing intermediate reasoning steps in written form, self‐explanation may have functioned as a form of offloading, reducing the need to process several interacting elements simultaneously. This sequential and externally supported processing likely facilitated the integration of relational information, which is key for the abstraction of underlying problem schemas (Sweller, 1988).

We detected the indirect effect only for mean levels of fluid intelligence. This pattern may reflect a non‐linear relationship across the aptitude continuum. Early aptitude–treatment interaction research typically compared high‐ and low‐ability groups and reported that learners with higher fluid intelligence benefit more from less structured instructional environments (Snow, 1992; Snow & Lohman, 1984). Learners with higher reasoning ability may spontaneously process relational information, such that worked examples exert their influence more directly and independently of observable differences in self‐explanation quality. The engagement activity adds another layer of structure these learners do not need. While adding another layer of structure, learners with lower reasoning ability may lack sufficient cognitive resources to generate high‐quality self‐explanations even when prompted, limiting the mediating role of self‐explanation. For learners with medium levels of fluid intelligence, however, cognitive resources are sufficient to benefit from engaging in high‐quality self‐explanations, yet not so high that the necessary processing of the worked example occurs automatically. In this range, self‐explanation quality becomes a decisive mechanism linking worked examples to learning outcomes.

### Theoretical and practical implications

The present findings yield two central theoretical contributions. First, they indicate that worked examples exert both direct and indirect effects on learning, and that the relative importance of both these pathways depends on learners' cognitive aptitudes. Consistent with prior work (Bichler et al., 2020, 2022), we observe evidence for a direct benefit of worked examples. At the same time, we demonstrate that an indirect pathway via self‐explanation quality (Atkinson et al., 2000) operates for specific aptitude constellations. Thus, the worked example effect is not driven by a single, uniform mechanism. Rather, the mechanism itself varies as a function of individual differences.

Second, our findings refine assumptions about how scaffolds interact with cognitive architecture. While instructional theory has long emphasized aligning instruction with cognitive architecture (Sweller et al., 2019), our results differentiate which component of that architecture appears most relevant. It appears that worked examples primarily reduce demands on shifting ability rather than on storage capacity, as previously hypothesized (Bichler et al., 2020; Schwaighofer et al., 2016; van Gog & Rummel, 2010).

These findings have both instructional design and teaching implications. In terms of design, instructional scaffolds should be structured to reduce demands on learners' ability to flexibly shift between information elements, goal states, and operators. In terms of teaching, worked examples should be paired with self‐explanation prompts for learners with lower prior knowledge, lower working memory capacity, or average reasoning ability. Information about cognitive abilities is often unavailable to instructors, but when such information is accessible or can be reasonably estimated, engaging learners in self‐explanation of worked examples is likely to maximize learning benefits. Further, instructors often assess knowledge levels (Bach et al., 2025), and this information can be used to tailor instruction, ideally by coupling worked examples with self‐explanation for learners with less prior knowledge.

### Limitations and future steps

By modelling all three components—scaffold, engagement activity, and learner aptitudes—our conditional process analysis provides a comprehensive view of how instructional support impacts learning and produces results that are both practically relevant and ecologically valid. However, modelling the complexities of real‐life learning also leads to complex statistical models that require large sample sizes for high statistical power (Aguinis, 1995; Schoemann et al., 2017). Thus, our studies fall short of the desired statistical power, limiting our conclusions by reducing the certainty that the findings are not spurious. A priori power for such models cannot be determined analytically. Instead, it requires simulation, which is computationally demanding and not yet common practice (Preacher & Sterba, 2019; Schoemann et al., 2017). Bringing even complex power estimations into common practice is a key challenge that must be addressed to identify, investigate, and analyse complex learning with greater rigour in the future.

Further, we analysed each core cognitive aptitude individually. Real‐world learning in scaffolded environments is shaped by the combined influence of multiple aptitudes (Schwaighofer et al., 2017), a complexity not reflected in our models. However, practically, the precise interplay of several cognitive aptitudes and instructional scaffolds might not be that important. Our results suggest that worked examples serve as effective support for learners across different cognitive profiles, whether through their design or in combination with self‐explaining. Offering both the scaffold and the engagement activity increases the chances that a diverse range of learners will benefit from the instruction.

Still, future research could investigate whether one aptitude can compensate for others. For example, individual differences in WMC might be meaningless, because they are compensated by individual differences in long‐term memory knowledge structures (Paas & Sweller, 2014). Additionally, it has been suggested that knowledge structures may reduce the reliance on executive functions (Sweller, 2004), which might be why cognitive aptitudes such as shifting are less prominently discussed in instructional theory (for a critical stance see Schüler et al., 2011). Such investigations could provide insights into the relative importance of individual aptitudes. This would help sharpen principles for instructional design, identifying which cognitive aptitudes should be prioritized for which group of learners.

Finally, our investigation concentrated exclusively on cognitive aptitudes, which represent only a subset of the broader spectrum of aptitudes relevant to learning. Learning aptitudes also encompass metacognitive, motivational, affective, and socio‐cultural dimensions. Future research could benefit from examining how conditions might influence the prioritization of these various aptitudes in different learning contexts (Kalyuga, 2026).

## CONCLUSION

This investigation into the interplay of scaffolds, engagement activities, and learner aptitudes reveals that learners benefit from worked examples in different ways depending on their prior knowledge, general reasoning ability, and basic cognitive functions. For instance, learners with lower prior domain knowledge, working memory capacity (WMC), or average reasoning ability tend to benefit from self‐explanation, as this engagement activity enhances the effect worked examples have on learning gains for these learners. Meanwhile, those with lower shifting ability benefit directly from worked examples, as these reduce shifting demands; learners can focus on abstracting solution strategies for ill‐defined problems or on structural features of the problem. Similarly, it seems that learners with higher reasoning ability can already extract the necessary insights directly from worked examples, while learners with lower reasoning ability may lack the capacity to benefit from the self‐explanation process. Taken together, these findings highlight that scaffolds can provide direct benefits or benefits mediated through engagement activities depending on individual aptitudes, and underscore shifting ability as a key component of human cognitive architecture central to effective instructional design.

## AUTHOR CONTRIBUTIONS

**Sarah Bichler:** Conceptualization; data curation; formal analysis; investigation; methodology; project administration; visualization; writing – original draft. **Matthias Stadler:** Supervision; writing – review and editing. **Markus Bühner:** Resources; software; writing – review and editing. **Samuel Greiff:** Supervision; writing – review and editing. **Frank Fischer:** Conceptualization; funding acquisition; resources; supervision; software; writing – review and editing.

## FUNDING INFORMATION

Research reported in this manuscript was supported by the Elite Network of Bavaria (K‐GS‐2012‐209).

## CONFLICT OF INTEREST STATEMENT

The authors report there are no competing interests to declare, except that publication in peer reviewed journals is crucial for advancing the academic career of (some) authors.

## DECLARATION OF GENERATIVE AI AND AI‐ASSISTED TECHNOLOGIES IN THE WRITING PROCESS

During the preparation of this work the author(s) used OpenAI (2024) ChatGPT‐4 [Large language model], https://chat.openai.com/chat to refine and improve writing (grammar checks, expression, conciseness, shortening). After using this tool/service, the author(s) reviewed and edited the content as needed and take(s) full responsibility for the content of the publication.

## Data Availability

The data that support the findings of this study are openly available in OSF at https://doi.org/10.17605/OSF.IO/CZSRP.
